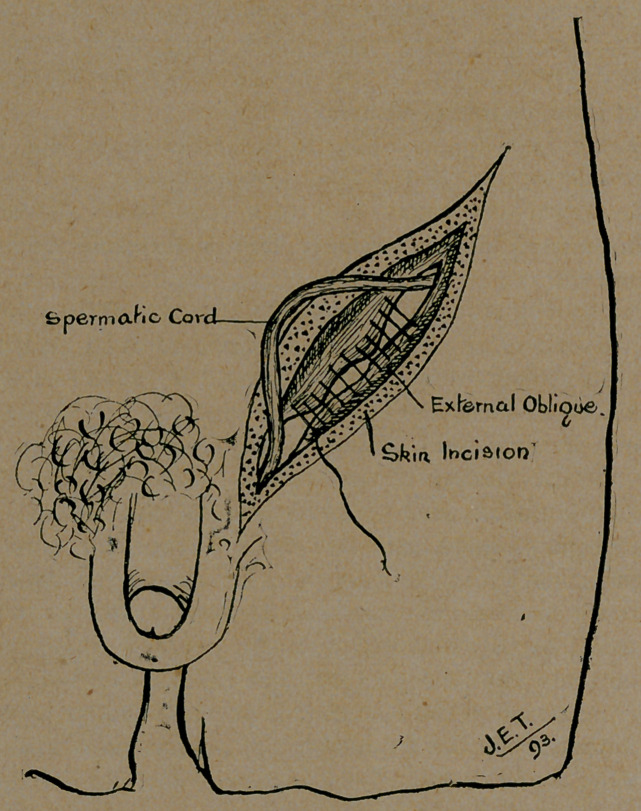# Radical Cure for Inguinal Hernia

**Published:** 1893-05

**Authors:** James E. Thompson

**Affiliations:** M. B. B. S. Lond., F. R. C. S. Eng.; Galveston


### DANIEL'S TEXAS MEDICAL JOURNAL.

ESTABLISHED JULY, 1885.

PUBLISHED MONTHLY.—SUBSCRIPTION $2.00 A YEAR.

Vol. VIII. AUSTIN, MAY, 1893. No. 11.

### Original Contributions.

For Daniel's Texas Medical Journal:

### RADICAL CURE FOR INGUINAL HERNIA.

BY JAMES E. THOMPSON, M. B. B. S. LOND., F. R. C. S. ENG.

[Delivered Before the Galveston Medical Society.]

THE history of the operation of radical cure for inguinal hernia is replete with interest, and shows to a marked degree the evolution of surgical thought and the pliability and adaptability of surgical intellect. Engraved upon it are the imprints of all the great surgical advances, and no branch, save abdominal surgery, of which it is now a part, owes more to the teachings of the great Lister.

The older methods of operating were crude in the extreme, and one and all reflect most vividly the uppermost thought in the surgeon's mind, intense fear lest the peritoneal cavity should be tampered with.

Sir Astley Cooper* writing in 1827, says that a ligature applied around a part of the peritoneum must inflame it; and, as this membrane is continued without interruption along the sac into the cavity of the abdomen, the inflammation will follow the same course and expose the patient's life to hazard.

A slight review of the older methods will repay us for our trouble.

*Anatomy and surgical treatment of abdominal hernia. London, 1827.

Direct pressure on the orifice of external abdominal ring was one of the earliest methods employed to secure obliteration of the hernial sac.

A conical lint pad was inserted into the ring and kept there by an elastic truss; the supine position was maintained for about four weeks, at which period suppuration was established. By some surgeons irritating applications, such as turpentine and cantharides were applied underneath the pad.

The treatment by the actual cautery dates from the Alexandrian school, and is mentioned by Paulus Ægineta.

By this method the tissues were gradually burnt away and the sac eventually destroyed.

† Various escharotics, such as caustic-potash, arsenic, lime and sulphuric acid have been used from time to time for the same purpose.

Ligature of the sac and stitching the sac by a number of sutures was the first real advance.

It was done in two ways, either through an incision which laid bare the sac, or sub-cutaneously, the threads being passed around the isolated sac by a needle, the point of exit of the instrument coinciding with the point of entry. In fact, an operation exactly similar to one commonly employed for varicocele.

Invagination of a plug of skin was tried by Dzondi, Jameson and Gerdy. Gerdy's operation deserves some description.

The scrotum was firmly invaginated into the inguinal canal by the tips of the left forefinger. A threaded needle was passed along the finger and thrust through the invaginated scrotum, external oblique, and abdomina skin. One end of the thread was pulled out and the needle withdrawn. Still threaded, it was introduced a second time, piercing the same structures nearer the middle line of the body; the needle was withdrawn and the loop cut. A plug of plaster was then fastened to the scrotal ends, and this was firmly drawn up into the mouth of the inguinal canal by hauling on the abdominal threads.

Signorini invaginated the skin of the scrotum and fixed it in situ by hare-lip pins, around the ends of which silk was fastened in the form of a figure of 8. ‡ Bonnet performed an operation by which the hernial sac was obliterated subcutaneously. A num-

---

† South's Chelius, p. 282, Vol. 1.

‡ Journal des Connaissances Medico-Chirugical. July, 1836, quoted in South's Chelius, loc. cit.

ber of pins were passed between the sac and the structures of the cord, the tissues being compressed by corks thrust along the pins which were then bent to prevent the corks slipping. Wutzer had a most ingenious apparatus. It consisted of a box-wood cylinder about three inches long, perforated from end to end, and containing a curved steel tube through which a needle could be thrust.

The cylinder was firmly pressed into the inguinal canal, carrying with it invaginated scrotum. The needle was then passed through the tube and made to perforate the external oblique and skin, showing itself on the abdominal wall. A concave box-wood case was then passed over the projecting point of the needle and was firmly fixed to the other end of the cylinder by a screw apparatus. By this means the tissues were firmly compressed. This operation was attended by great success in Wutzer's hands. *Belmas attempted to excite inflammation in the hernial sac by introducing gold-beater's skin. A similar method has lately been introduced, by which various substances, such as alcohol and tincture of oak-bark are injected into the tissue around the circumference of the hernial aperture. In the hands of Heaton, Warren and Reetley, this has been attended with a fair amount of success. A new era in the operative treatment was opened with the publication of Wood's treatise on hernia in 1863.

His operation was conducted on true anatomical principles, and was the result of long training, both in the dead house and in the operating theatre.

An incision large enough to admit the forefinger and needle was made in the scrotum about half an inch below the external abdominal ring; the margins of the incision were freed from the subcutaneous tissues for some little distance at the upper part, to enable the operator to invaginate the scrotal tissues into the inguinal canal. A needle was now carefully passed along the invaginating forefinger (the cord being carefully isolated), and the edge of the conjoined tendon felt for. This was pierced from within outwards, the needle passing through the overlying external oblique and skin. It was then threaded with silver wire and withdrawn, one end of the wire being left emerging from the abdominal puncture. The unthreaded needle was then passed as

---

* Revue Medicale, March, 1838. Clinique des Hopitaux, August 21, September 11, 1839.

before, but this time through the outer pillar of the external inguinal aperture, emerging through the skin at the same point as before. It was then threaded and withdrawn, carrying with it the other end of the wire, a loop remaining outside the abdominal puncture. The needle was then released, passed through the outer pillar of the ring, underneath the hernial sac, there threaded with the inner end of the wire and withdrawn. The same manucœuvre was done on the inner side, the outer wire being drawn under the hernial sac and through the internal pillar. A pad of gauze was fastened in the loop outside the skin puncture, and the scrotal ends of the wire drawn tight and twisted.

By this procedure the sac was compressed and the pillars of the ring laced together like a boot. The wire was allowed to remain in situ for about ten days; the loop was then cut and each end withdrawn.

In his later operations, Wood used catgut or kangaroo tendon and buried the sutures.

The next operation that came into vogue was Spanton's. A portion of the subcutaneous tissue of the scrotum was invaginated and then a cork-screw-like instrument was introduced through the skin at the level of the internal ring. By twisting the instrument the point passed alternately through the pillars of the ring and the same structures were secured as in Wood's operation.

It has been incidentally remarked that perhaps this gentleman was more accustomed to use a cork-screw than a knife.

†Macewen brought forward the next advance. He, recognizing the fact that the structures forming the external inguinal aparture have but a small influence in preventing the recurrence of the rupture, sought to close up the inguinal canal in a valvular manner.

The external ring was clearly exposed by an incision, the hernial sac separated in toto up to its highest point from the structures of the cord. A knotted silk thread was then passed through the lower end of the sac and carried alternately from side to side, until the external ring was reached. The thread was then passed under the edge of the external oblique and made to pierce that muscle at the level of the internal ring. By pulling on the thread, the sac was rolled up tightly into the original

---

†British Medical Journal, Dec. 10, 1882.

canal, there forming a plug. A thread was then passed through the edge of the conjoined tendon and this was pulled outwards and downwards and attached to the outer pillar of the external ring. The two pillars of the external ring were then united.

†Barker has another modification. He ligatures the neck of the sac, cuts it free and pulls it up under cover of the external oblique. He then united accurately the pillars of the external ring, leaving sufficient space for the spermatic cord. The greater part of the sac is left undisturbed in the scrotal tissues.

In 1890 *Bassini published an account of an operation for radical cure of inguinal hernia, which seems to fulfil all necessary indications for success.

The steps of this operation may be briefly summarized as follows:

1. The skin incision extends the whole length of the inguinal canal.

2. The external oblique is slit from the external ring up to the level of the internal ring.

3. The sac of the hernia is carefully separated from the strictures of the cord, ligated and removed.

4. The spermatic cord is lifted from its bed, and the upper border of Poupart's ligament and the lower border of internal oblique and conjoined tendon exposed by a few strokes of the scalpel.

5. The lower arched border of internal oblique and conjoined tendon are united to Poupart's ligament by a continuous suture.

6. The cord is then placed in the new inguinal canal.

7. The two edges of external oblique are then united over the cord by a continuous suture—a sufficient space being left at the inner end of the exit of the cord.

8. The skin incision is united by a continuous suture.

The method I have adopted in the cases I shall presently report is a modification of Bassini's. I thought up to a few weeks ago that the innovation was my own, but I have reason to believe that Halstead has employed it.

The earlier steps of the operation are the same as those of Bassini's, but in the last stage the edges of the external oblique are united behind the cord, thus closing firmly and completely the inguinal canal.

---

†British Medical Journal, Dec. 3, 1887.

*Archiv f. Klin. Chir., 1890, t. XL, p. 429.

The cord emerges from the external oblique at the level of the internal ring and passes thence *subcutaneonsly* to the scrotum.

In this historical sketch of the various operations I have necessarily omitted many operations, some purposely, because they have been abandoned by their authors, and others because they failed to throw any light on the development of the operation.

The various steps of the operation are worthy of some consideration. The skin incision in the majority of cases reaches from the level of the internal ring (the middle of Poupart's ligament) to a point half an inch below the external ring.

It is quickly deepened until the external oblique is reached; all bleeding points being at once secured with artery forceps and tied with catgut. The external ring is clearly defined and a director thrust through it, underneath the external oblique, to the internal ring. The aponeurosis is now slit up in the line of the inguinal canal. The two edges of the aponeurosis are retracted, and the hernial sac carefully separated from the cord.

Sometimes this can be easily accomplished, but, occasionally, and this is the rule in cases of congenital hernia, the separation is exceedingly difficult. It is my rule always to open the sac in situ and carefully explore the margins of the abdominal ring to make sure that no intestinal structures are adherent in this situation. If omentum is adherent to the sac it is ligated and removed. Often it can be peeled from the surface of the sac and ligatured "en masse" higher up, but more often it is better to ligate in successive steps, each vessel being secured separately.

It is rarely necessary to pull down and remove normal omentum as Championnière advises.

Leaving omentum as a plng in the orifice of the sac is a procedure that merits the strongest condemnation; as it has been clearly shown that it may lead to a fatal issue afterwards by forming a band under which a loop of intestine may become strangulated.

After having reduced the contents, the sac is now separated from the structures of the cord and ligatured at its highest point.

During the separation due care should be taken of the vas deferens and the spermatic artery, although the last named structnre can, in most cases, be cut through with impunity without danger to the vitality of the testicle. The stump of the ligatured sac is pushed well up under cover of the internal oblique.

Now comes a question: Are we to remove the remainder of the sac or leave it in the scrotum? I lay down no definite rule here, each case resting on its own merits. If the sac is not very adherent I usually remove it entirely, but if its connections with the tissues of the cord are very close I allow it to remain.

The next step in the operation is probably the most important. If a normal body be examined, the transversalis fascia, which forms the posterior wall of the inguinal canal for its outer two-thirds, is found to be a particularly strong structure. It is unyielding to a degree, and its integrity is a great factor in the normal strength of the abdominal wall at this point.

The case I shall show to-night exhibited to a marked degree the importance of this fascia. On opening the inguinal canal and drawing aside the cord, the abdominal contents bulged forward through the space normally walled in by the transversalis fascia; the swelling thus formed had a depression in its centre caused by the deep epigastric artery. There was no neck to the sac; there were, in fact, two sacs, one inside and the other outside the deep epigastric artery.

I believe that weakness of fascial structure is the greatest factor in the predisposing causes of acquired hernia.

The material for sutures has been a source of great anxiety. Up to a few months ago I had perfect faith in silk sterilized by being boiled for an hour in a five per cent. solution of carbolic acid. It has, however, played me such sad pranks that I have abandoned it and use specially prepared catgut kept in oil of juniper and spirit. The change has been a salutary one and I have no reason to regret it. My skin sutures are also of catgut and are continuous, the wound being entirely closed, no drainage being necessary. The wound is irrigated at each successive step with 1-2000 solution of corrosive sublimate.

The skin incision is dusted with iodoform and dressed with iodoform gauze and sublimate cotton. A firm spica is put on and the patient carefully removed to bed. In reference to after treatment nothing need be said, except that perfect rest is an absolute necessity. On no account should the patient get up under two weeks, and it is even better to keep him in bed for three or four weeks.

For the first twenty-four hours little is given except milk, and then, if everything is going well, ordinary diet is given. Unless pain or elevation of temperature requires it, the dressing is not changed for seven days, when the sutures are removed. During the past three months I have operated successfully on three cases by this method. A slight resumé will be interesting.

Case 1. John K., Irishman, 57 years of age, had suffered from a reducible right inguinal hernia for two years. In addition to this, he was a perfect pathological curiosity shop, having a contracted finger, a small ventral hernia above the umbilicus, and an encysted hydrocele of the right spermatic cord. As the rupture had been troubling him for some time I decided to operate.

On incising what I thought was the hernial sac, I was surprised to find a blind cavity containing a clear fluid. Recognizing that this might be a hydrocele of the cord associated with a hernia, I cut through the posterior wall, and after incising two serous membranes, entered the true hernial sac. This was explored with the finger; the margins of the internal ring were free, and I dissected out the whole sac, ligating it at the upper end. I completed the operation as above described, leaving the spermatic cord under the skin.

Case 2. A negro, aged 40, was brought to me, suffering from a reducible left inguinal hernia, associated with a syphilitic testicle on the same side. The hernia had been present about five years, and had given rise to no urgent symptoms.

A gummatous ulcer, the size of a dollar, and a quarter of an inch deep, still existed at the lower end of the testicle; and there was an old scar connected with the lower part of the right testicle.

Excision of the testicle and radical cure of hernia were decided on. The operation was conducted as before but the cord and sac were ligated in the same knot, as it was found almost impossible to separate them. The patient made a successful recovery.

Case 3. Edward C., white, aged 45. Presented a double bubonocele. The right hernia had been giving much trouble, so an operation was decided upon.

The operation was conducted as before, but no incision was carried into the scrotum. On slitting up the external oblique, the sac of the hernia was found to have no neck, but the yielding transversalis facia was pushed bodily in front of it. The union of internal oblique and conjoined tendon with Poupart's ligament completely obliterated the tendency to bulging. The cord was brought out subcutaneously. The patient made a quick recovery.

My method of procedure before was a combination of Macewen's and Barker's, and with this I obtained excellent results; nevertheless, I consider that this method of buttressing the structures is more scientific and shows the best results.

Unfortunately, however, I have been unable to follow the cases, and as the men were unable on discharge to provide themselves with trusses, I am afraid that some degree of recurrence is only a question of time. Altogether, I have operated on twenty cases with no mortality; and in the few I have been able to follow, for one year only after the operation, there was no recurrence.

In reference to recurrence, I think that no operation will ensure freedom without the sincerest co-operation on the part of the patient.

A well fitting form of abdominal support should be worn for at least two years after the operation, and should be removed only when the patient is in a recumbent attitude.

The statistics of the operations for radical cure of inguinal hernia are as follows:

| | Operations | Deaths |
|---|---|---|
| Bassini | 262 | 1 |
| Championnière | 254 | 2 |
| Schede | 165 | 2 |
| Banks | 106 | 0 |
| Park | 115 | 0 |
| Marcy | 115 | 0 |
| IN THE PRE-ANTISEPTIC ERA. | | |
| Wood | 339 | 7 |

This gives a mortality in the hands of the best operators of less than ½ per cent.

Now contrast this with the following list of operations for stangulated hernia taken from the case books of the Manchester Royal Infirmary. During nine months, forty-nine operations came under my personal observation, with fifteen deaths, giving a mortality of almost 30 per cent. Of these the analysis reveals.

| | Operations. | Deaths. |
|---|---|---|
| Inguinal hernia . . . . . . . . . . . . . . | 20 | 7 |
| Femoral hernia . . . . . . . . . . . . . . | 15 | 4 |
| Umbilical hernia . . . . . . . . . . . . . | 5 | 4 |

Giving a mortality of

| | |
|---|---|
| Inguinal . . . . . . . . . . . . . . . . . . . . | 24.4% |
| Femoral . . . . . . . . . . . . . . . . . . . . | 20 % |
| Umbilical . . . . . . . . . . . . . . . . . . . | 80 % |

Amongst these operations there were two enterectomies, the one for umbilical hernia, the other for inguinal hernia. Both were fatal. In the face of these alarming statistics, and there is here nothing hidden, can we view without concern the existence of hernia in any patient?

Is not the difference between ½ per cent. and 20 per cent. sufficient reason for every surgeon to urge most strenuously the performance of an operatation for radical cure?

Every afflicted man may have his turn sooner or later, and the enormous difference between the mortality of strangulated and non-strangulated hernia, makes it incumbent on every surgeon to give his patient a chance of avoiding the terrible risks which are daily threatening his life, and which may overtake him when skilled assistance is unattainable.

---

For Daniel's Texas Medical Journal.

## Figures and Tables

**Figure f1:**
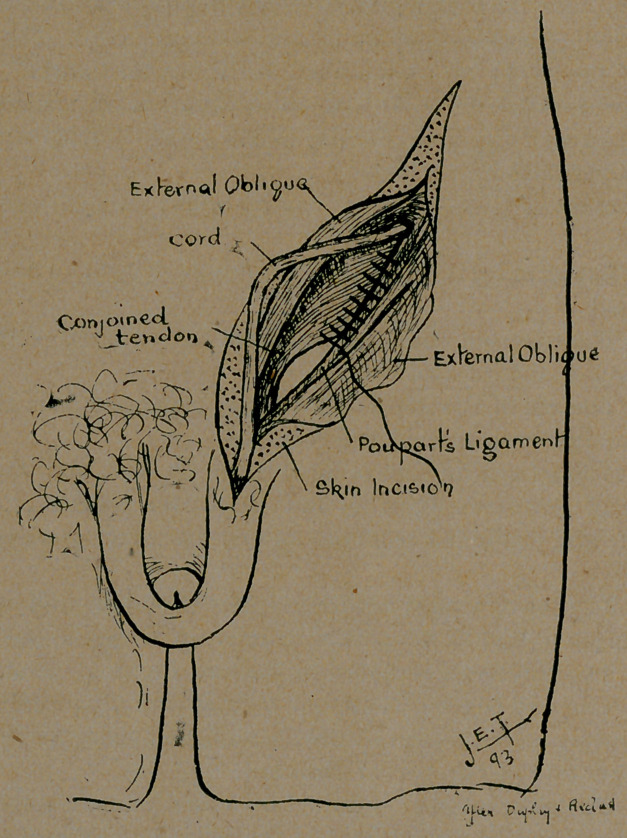


**Figure f2:**